# Supplementary material for: Rapamycin rejuvenates oral health in aging mice
Source: eLife. 2020 Apr 28;9:e54318. doi: 10.7554/eLife.54318 (PMC7220376; doi:10.7554/eLife.54318)
Supplement: Transparent reporting form [file elife-54318-transrepform.pdf]

eLIFE
1st Floor
24 Hills Road
Cambridge CB2 1JP, UK
P 01223 855340
W elifesciences.org
T @elife

eLife's transparent reporting form

We encourage authors to provide detailed information *within their submission* to facilitate the interpretation and replication of experiments. Authors can upload supporting documentation to indicate the use of appropriate reporting guidelines for health-related research (see EQUATOR Network), life science research (see the BioSharing Information Resource), or the ARRIVE guidelines for reporting work involving animal research. Where applicable, authors should refer to any relevant reporting standards documents in this form.

If you have any questions, please consult our Journal Policies and/or contact us: editorial@elifesciences.org.

Sample-size estimation

- You should state whether an appropriate sample size was computed when the study was being designed
- You should state the statistical method of sample size computation and any required assumptions
- If no explicit power analysis was used, you should describe how you decided what sample (replicate) size (number) to use

Please outline where this information can be found within the submission (e.g., sections or figure legends), or explain why this information doesn't apply to your submission:

We structured our design with measurements of periodontal bone as the primary endpoint. Power analyses were performed using data gathered from previous studies to estimate effect size for age and treatment for changes in periodontal bone. In order to reliably detect a 20% change between treatment, we estimated groups of n=10 animals should be used for each group. This cohort size was used at each location independently, as described in the materials and methods. No power analysis was performed for other assessments, including inflammatory markers by Western blot and microbiome changes.

Replicates

- You should report how often each experiment was performed
- You should include a definition of biological versus technical replication
- The data obtained should be provided and sufficient information should be provided to indicate the number of independent biological and/or technical replicates
- If you encountered any outliers, you should describe how these were handled
- Criteria for exclusion/inclusion of data should be clearly stated
- High-throughput sequence data should be uploaded before submission, with a private link for reviewers provided (these are available from both GEO and ArrayExpress)

Please outline where this information can be found within the submission (e.g., sections or figure legends), or explain why this information doesn't apply to your submission:

As described in the results and methods sections, two independent cohorts of mice were used for this study and housed at different locations. Sample sizes of each cohort are provided in the results and methods section. All of the analyses were performed in a blinded fashion and no data were excluded as outliers or for other reasons. Sequence data has been made publicly available as indicated in the manuscript.

eLIFE
1st Floor
24 Hills Road
Cambridge CB2 1JP, UK
P 01223 855340
W elifesciences.org
T @elife

Statistical reporting

- Statistical analysis methods should be described and justified
- Raw data should be presented in figures whenever informative to do so (typically when N per group is less than 10)
- For each experiment, you should identify the statistical tests used, exact values of N, definitions of center, methods of multiple test correction, and dispersion and precision measures (e.g., mean, median, SD, SEM, confidence intervals; and, for the major substantive results, a measure of effect size (e.g., Pearson's r, Cohen's d)
- Report exact p-values wherever possible alongside the summary statistics and 95% confidence intervals. These should be reported for all key questions and not only when the p-value is less than 0.05.

Please outline where this information can be found within the submission (e.g., sections or figure legends), or explain why this information doesn't apply to your submission:

Detailed descriptions of the statistical methods used are provided in the materials and methods section.

(For large datasets, or papers with a very large number of statistical tests, you may upload a single table file with tests, Ns, etc., with reference to sections in the manuscript.)

Group allocation

- Indicate how samples were allocated into experimental groups (in the case of clinical studies, please specify allocation to treatment method); if randomization was used, please also state if restricted randomization was applied
- Indicate if masking was used during group allocation, data collection and/or data analysis

Please outline where this information can be found within the submission (e.g., sections or figure legends), or explain why this information doesn't apply to your submission:

Aged animals were randomly assigned to either vehicle control or rapamycin treatment cohorts at both sites. Experimenters were blinded to treatment group until all analyses were completed.

Additional data files ("source data")

- We encourage you to upload relevant additional data files, such as numerical data that are represented as a graph in a figure, or as a summary table
- Where provided, these should be in the most useful format, and they can be uploaded as "Source data" files linked to a main figure or table
- Include model definition files including the full list of parameters used
- Include code used for data analysis (e.g., R, MatLab)
- Avoid stating that data files are "available upon request"

Please indicate the figures or tables for which source data files have been provided:

All data used in the development of this manuscript and the supplemental material are available in the manuscript or the supplemental materials or upon request. Bioinformatic scripts and microbiome data used in the analysis and generation of figures for this manuscript are available on the McLean Lab GitHub repository: https://github.com/kkerns85/Rapamycin_rejuvenates_oral_health_in_aging_mice.git. In addition, a web version of the R Markdown is available on Rpubs: https://rpubs.com/kkerns85/Rapamycin_Rmrkdown. The V4-16S rDNA sequences in raw format, prior to post-processing and data analysis, have been deposited at the European Nucleotide Archive (ENA) under study accession no. PRJEB35672.

eLife Sciences Publications, Ltd is a limited liability non-profit non-stock corporation incorporated in the State of Delaware, USA, with company number 5030732, and is registered in the UK with company number FC030576 and branch number BR015634 at the address 1st Floor, 24 Hills Road, Cambridge CB2 1JP | August 2014
